# Hip stability after total hip arthroplasty predicted by intraoperative stability test and range of motion: a cross-sectional study

**DOI:** 10.1186/s12891-018-2289-y

**Published:** 2018-10-15

**Authors:** Hiromasa Tanino, Tatsuya Sato, Yasuhiro Nishida, Ryo Mitsutake, Hiroshi Ito

**Affiliations:** Department of Orthopaedic Surgery, Asahikawa Medical University, Midorigaoka-Higashi 2-1-1-1, Asahikawa, 078-8510 Japan

**Keywords:** Dislocation, Total hip arthroplasty, Range of motion, Hip

## Abstract

**Background:**

Dislocation continues to be a common complication following total hip arthroplasty (THA). A larger intraoperative range of motion (ROM) is believed to minimize dislocation risk, and intraoperative stability tests have been used to assess the ROM. However, it is not clear whether or not intraoperative stability tests can predict hip stability after THA. It is also unclear which angles are required in intraoperative stability tests. We investigated the usefulness of intraoperative stability tests, and other risk factors to predict hip stability after THA.

**Methods:**

Patients operated by single surgeon at one hospital from June 2009 to December 2013 were evaluated. This study included 185 hips with 32 mm metal femoral head. The range of internal rotation with 90° hip flexion (IR angle) was measured as an intraoperative stability test. The variables studied as risk factors included age, height, weight, gender, cerebral dysfunction, preoperative diagnosis, history of previous hip surgery, and IR angle.

**Results:**

Mean IR angle was statistically different between patients with dislocation and patients without dislocation (59.5° vs 69.6°: *p* = 0.006). Cerebral dysfunction and a history of previous hip surgery were statistically related with prevalence of dislocation (*p* = 0.021, and *p* = 0.011). The receiver-operating characteristic curve analysis suggested that the cutoff points for IR angle were 51° and 67°. Dislocation rate in larger IR angle group was significantly lower than the rate in smaller IR angle group when patients were divided by 51° (*p* = 0.002). Logistic regression analyses showed that significant risk factors were cerebral dysfunction (OR: 5.3 (95%CI 1.1–25.9); *p* = 0.037), history of previous hip surgery (OR: 8.6 (95%CI 1.2–63.0); *p* = 0.035), and IR angle (OR: 10.4 (95%CI 1.9–57.1); *p* = 0.007).

**Conclusions:**

The results showed that intraoperative stability test, especially the IR angle, was a useful method to predict hip stability after THA, and a larger intraoperative ROM reduced the likelihood of dislocation. 51° and 67° were indicated as cutoff points for IR angle. Cerebral dysfunction and a history of previous hip surgery are also risk factors for the incidence of dislocation after THA.

**Trial registration:**

This is a retrospective study, not a clinical trial defined by the World Health Organization (WHO).

## Background

Dislocation continues to be a common complication following total hip arthroplasty (THA). In a recent study of 51,345 revision hip arthroplasties in the United States, dislocation was the most common cause of revision (22.5%), and was higher than both infection and aseptic loosening [1]. In the Australian Orthopaedic Association Registry, dislocation was reported as the reason for revision in 21.6% of cases, which was next after loosening [2]. Many factors affect the prevalence of dislocation after THA, including soft-tissue laxity, surgical approach, component position, patient factors, and component designs [3]. A larger intraoperative range of motion (ROM) is believed to minimize dislocation risk, and intraoperative stability tests have been used to assess the ROM [4]. Many in vitro studies have investigated the ROM of the hip, impingement, and dislocation mechanisms, and they reported the factors related to ROM, including component position, head diameter, and component design [5]. However, it is not clear whether or not intraoperative stability tests can predict hip stability after THA. It is also unclear which angles are required in intraoperative stability tests for a stable hip after THA because different angles have been indicated as an acceptable ROM [4, 6–9]. This study included patients with one metal femoral head diameter and evaluated the effect of intraoperative ROM on dislocation rates after THA. We also evaluated the combined effect of other risk factors in conjunction with intraoperative ROM. Our hypothesis was that intraoperative stability tests would be effective to indicate hip stability after THA, and that a large intraoperative ROM would reduce the likelihood of dislocation after THA.

## Methods

### Study population

**Fig. 1:**
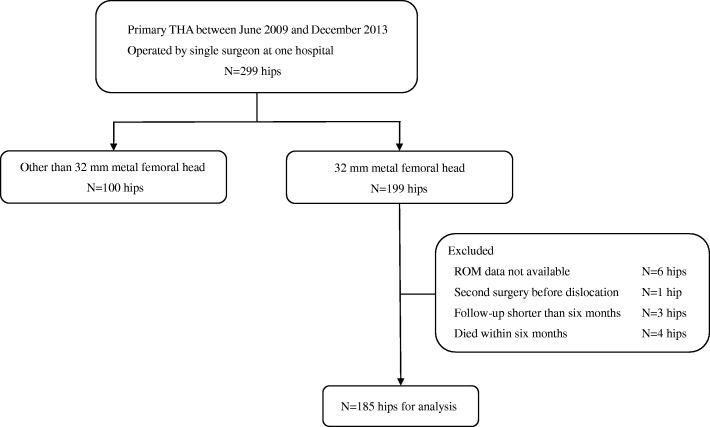
Flowchart depicting patient inclusion and exclusion in the cross-sectional study

Between June 2009 and December 2013, the senior surgeon performed primary THA in 274 consecutive patients (299 hips) at Asahikawa Medical University Hospital. For inclusion in the analysis, the following were required: primary THA, single surgeon at one hospital, intraoperative ROM data available, no second surgeries before the first dislocation event, follow-up longer than 6 months, and a 32 mm metal femoral head had been used. A 32 mm metal femoral head was used in 185 patients (199 hips) out of an initial 274 patients. Fourteen hips were excluded (ROM data availability 6 hips, second surgery before the first dislocation 1 hip, short follow-up 3 hips, and died within 6 months 4 hips), thus 185 hips (171 patients) were included and analyzed in this study (Fig. 1). The average age was 64 years (range, 20–87 years), and height and weight averaged 153 cm and 58 kg, respectively. There were 128 women and 43 men, with 81 left and 104 right THAs. The average length of follow-up was 14 months (range, 6–44 months). The preoperative diagnoses were osteoarthritis in 146, osteonecrosis of the femoral head in 23, rheumatoid arthritis in 9, and femoral neck fracture in 7. The project was approved by the Institutional Review Board at our hospital (AMU 800).

### Surgery

All of the procedures were performed in the lateral decubitus position, using a posterolateral approach without posterior capsule or external rotator repair. All components were determined intraoperatively in a standard fashion based on preoperative templating, then placed into position. In general, patients older than age 60 received cemented femoral fixation, and younger patients had cementless femoral prostheses. The femoral prostheses used in this series included 149 cemented 4-U prostheses (Nakashima Medical Co., Japan) [10, 11] and 36 cementless prostheses (S-ROM; Depuy, IN or 4-U CLS; Nakashima Medical Co.). All of the acetabular components were cementless, including a 146 4-U cup (Nakashima Medical Co.), a 25 Trilogy cup (Zimmer, IN), and a 14 4-U CLS cup (Nakashima Medical Co.). The diameter of the metal femoral head was 32 mm with a polyethylene liner in all hips. A standard flat liner was used in 5 hips and an elevated liner in 180.

### Data collection

The variables studied as risk factors for dislocation after THA included age, height, weight, gender, cerebral dysfunction, preoperative diagnosis, and history of previous hip surgery. Cerebral dysfunction included mental confusion, dementia, and mental disorder at the time of surgery and dislocation; this was similar to another study [6]. Another category entitled *IR angle* was investigated as a measure of intraoperative stability. After all components were placed into position and removal of acetabular osteophytes and femoral neck remnants, the range of internal rotation with 90° hip flexion and 0° abduction/adduction (IR angle) was measured to determine the position of posterior dislocation, similar to Sultan et al. [12]. The femoral head was observed by the senior surgeon, while the hips were ranged by the assistant surgeon; the same assistant surgeon ranged all hips in this study. The point of instability was determined by direct visual inspection and was predefined as the point at which the head began riding out of the liner. A double-armed universal goniometer was used with one arm parallel to the floor and the other parallel to the tibia to determine hip rotation during measurement relative to the floor. The postoperative rehabilitation program was identical for all patients. Patients began ambulation on the first postoperative day and were allowed immediate full weight bearing using a walker or crutches. All patients included in this study were routinely followed-up at one clinic, and dislocation rates were obtained through clinic records.

### Statistical analysis

For continuous variables, the normality of the data was assessed using Shapiro-Wilk test, and statistical analysis was done using a nonparametric Mann-Whitney U test. Statistical analysis was done using a chi-square test for nominal variables. Receiver-operating characteristic (ROC) curve was applied to determine the optimum cutoff point for IR angle. The cutoff point was determined by the Youden index [13]. The area under curve (AUC) was also calculated from ROC curve. Logistic regression was performed using all eight variables; age, height, weight, gender, cerebral dysfunction, preoperative diagnosis, history of previous hip surgery, and IR angle. A *p* < 0.05 was considered significant. Statistical analyses were performed using SPSS Version 24 (SPSS Inc., IL).

## Results

Eleven patients (11 hips) sustained a hip dislocation resulting in a prevalence of 5.9%. Seven women and four men had 10 posterior dislocations and one anterior dislocation. Nine (82%) of the dislocations occurred during the first 3 months after surgery. Six patients had a single episode of dislocation and five had more than one dislocation. All dislocations were reduced without surgery. One of the 11 patients required revision of the cup, liner, and head because of instability.

Patient average age, height, weight and gender were not statistically different between patients with dislocation and patients without dislocation after THA (*p* = 0.195, *p* = 0.298, *p* = 0.197 and *p* = 0.249). There was a significant difference in the dislocation rates in the 29 patients (31 hips) classified as having cerebral dysfunction compared to the 142 patients (154 hips) with no cerebral dysfunction (16% vs 3.9%: *p* = 0.021). The preoperative diagnoses were categorized into 2 classes: 1) osteoarthritis, or 2) osteonecrosis of the femoral head, rheumatoid arthritis, and femoral neck fracture. Using this classification, there was no significant relationship between the class of diagnosis and the rate of dislocation (*p* = 0.419). A history of previous hip surgery was statistically related to prevalence of dislocation (33% vs 4.5%: *p* = 0.011) (Table 1).

**Table 1:** Studied Variables

Variables	Patients Without Dislocation (range)	Patients With Dislocation (range)	*p* Value
No. patients	160, 174 hips	11, 11 hips	
Average age (y)	64.4 (20–87)	59.1 (43–82)	0.195
Average height (cm)	152.7 (135.0–175.0)	156.8 (138.5–173.3)	0.298
Average weight (kg)	57.9 (34.0–91.0)	64.2 (36.0–93.0)	0.197
Gender			
Female	134	7	
Male	40	4	0.249
Cerebral dysfunction			
(+)	26	5	
(−)	148	6	0.021
Preoperative diagnosis			
OA	138	8	
ON, RA, Fx	36	3	0.419
History of previous hip surgery			
(+)	6	3	
(−)	168	8	0.011
IR angle	69.6° (37–95)	59.5° (45–75)	0.006

*OA* osteoarthritis, *ON* osteonecrosis of the femoral head, *RA* rheumatoid arthritis, *Fx* femoral neck fracture

No data missing

**Fig. 2:**
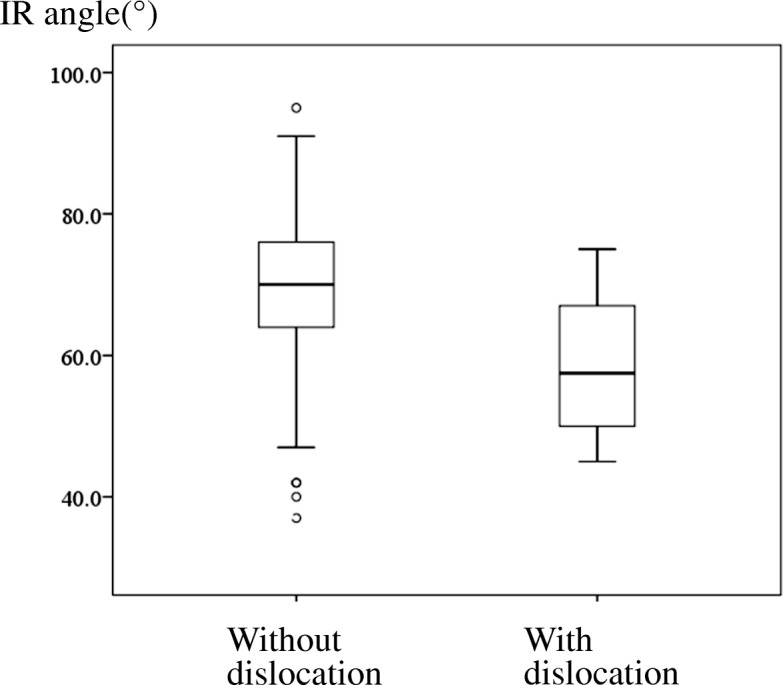
Box-plots of IR angles without dislocation and with dislocation

**Fig. 3:**
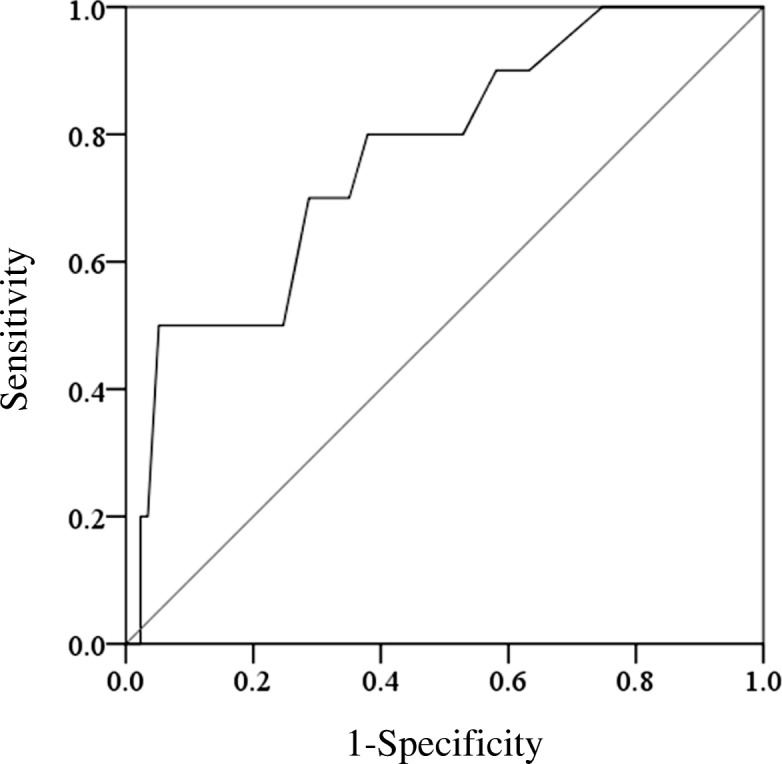
ROC curve of IR angle. The ROC curve analysis suggested the optimum cutoff point for IR angle was 51°, with a sensitivity of 0.5, a specificity of 1.0, a positive likelihood ratio of 9.7, and a negative likelihood ratio of 0.5. The AUC measured 0.8 (*p* = 0.004, 95%CI 0.6–0.9). When a sensitivity was 0.8, IR angle was 67°, with a specificity of 0.6, a positive likelihood ratio of 2.1, and a negative likelihood ratio of 0.3

The mean IR angle was 69.0° (range, 37–95°), and was statistically different between patients with dislocation and patients without dislocation after THA (59.5° vs 69.6°: *p* = 0.006) (Table 1). To investigate posterior stability, one patient with anterior dislocation was excluded from the subsequent analyses of IR angles. Mean IR angle was also statistically different between patients with posterior dislocation and patients who did not have a dislocation after THA (58.4° vs 69.6°: *p* = 0.004) (Fig. 2). The ROC curve analysis suggested the optimum cutoff point for IR angle was 51°, with a sensitivity of 0.5, a specificity of 1.0, a positive likelihood ratio of 9.7, and a negative likelihood ratio of 0.5. The AUC measured 0.8 (p = 0.004, 95%CI 0.6–0.9). When a sensitivity was 0.8, IR angle was 67°, with a specificity of 0.6, a positive likelihood ratio of 2.1, and a negative likelihood ratio of 0.3 (Fig. 3). Dislocation rate in larger IR angle group was significantly lower than the rate in smaller IR angle group when patients were divided by 51° (3.5% vs 33%: *p* = 0.002). For logistic regression analyses, all eight variables were used, and IR angle was used as a dichotomized variable (≥51° vs < 51°). Adjusting with age, height, weight, gender, and preoperative diagnosis, we observed that significant risk factors were the presence of cerebral dysfunction (OR: 5.3 (95%CI 1.1–25.9); *p* = 0.037), history of previous hip surgery (OR: 8.6 (95%CI 1.2–63.0); *p* = 0.035), and IR angle (OR: 10.4 (95%CI 1.9–57.1); *p* = 0.007) (Table 2).

**Table 2:** Association of Variables with the Incidence of Dislocation by Multiple Logistic Regression Analysis

Variables	Odds Ratio	95% Confidence Interval	*p* Value
Cerebral dysfunction	5.3	(1.1, 25.9)	0.037
History of previous hip surgery	8.6	(1.2, 63.0)	0.035
IR angle (ref. = ≥51°)	10.4	(1.9, 57.1)	0.007

## Discussion

We investigated the usefulness of intraoperative stability tests, and other risk factors to predict hip stability after THA. Intraoperative stability testing, especially IR angle, was a useful method to predict hip stability after THA, and a larger intraoperative ROM reduced the likelihood of dislocation after THA with a cutoff point of 51°. Cerebral dysfunction and a history of previous hip surgery are also risk factors for the incidence of dislocation after THA.

Robinson et al. reported IR angle was related to component position in their computational model, and Sultan et al. reported IR angle was related to the diameter of the prosthetic femoral head and the elevated liner intraoperatively [12, 14]. Recently, Bunn et al. investigated the relationship between intraoperative stability tests and high-risk activities for dislocation using computer modeling [15]. However, the validity of intraoperative stability tests in identifying risks for postoperative dislocation is unknown, as Bunn et al. stated [15]. Several different positions and tests have been used to investigate stability after THA. Harris described two critical positions to test the ROM: flexion plus internal rotation and extension plus external rotation [4]. The shuck test and drop kick test have been also used to test stability after THA [16]. The maximum flexion possible with the hip at 10° of adduction and 10° of internal rotation was used to test posterior stability in computational and experimental studies [17, 18]. Nadzadi et al. reported the kinematics of 5 positions associated with posterior dislocation and 2 positions associated with anterior dislocation [19]. It may be ideal to evaluate these 7 positions, but the authors feel it is too complex to implement during surgery. The results of this study showed that intraoperative stability test, especially the IR angle, was a useful method to predict posterior stability after THA. We found that cerebral dysfunction and history of previous hip surgery were also risk factors for the incidence of dislocation after THA, which is the same as in other studies [6].

It has not been clear which angles are required on intraoperative stability tests for stable hips after THA. Considerable variation in normal ROM exists among people and studies. The handbook of the American Academy of Orthopaedic Surgeons [20], which contains estimates of adult hip joint motion obtained from three referenced studies reported mean angles of internal rotation were 44°, 32°, and 33°. The prosthetic ROM must be larger than the normal ROM, as Widmer stated [21]. Harris reported that the limb after THA should flex to at least 110° and also go to 20° of internal rotation while at 90° of flexion without impingement [4]. Woolson et al. reported that hips after THA should have an intraoperative ROM of at least 90° of flexion combined with 30° of internal rotation without instability [6]. Sierra et al. reported 100° of flexion combined with 45° internal rotation as acceptable ROM [7]. Yoshimine used an IR angle of more than 30° as a moderate criteria of acceptable ROM conditions after THA in a computational study [8]. Recently, Lachiewicz and Soileau reported flexion past 90° and internal rotation of at least 60° as a stable hip using 36 mm or 40 mm femoral heads [9]. However, there were no explanations or reasons given for these acceptable ROMs. This study showed a larger intraoperative ROM reduced the likelihood of dislocation after THA, and 51° was indicated as a cutoff point for IR angle. Because it is important for surgeons to have an intraoperative stability test with a high sensitivity and our results showed IR angle was 67° when a sensitivity was 0.8, we also suggest 67° to be the severe criteria of cutoff point for IR angle. These cutoff points were larger than the normal ROM [20] and were also larger than the movements in commonplace maneuvers known to increase the risk for dislocation in THA [19]. Most modern THA systems provide the surgeon with a variety of options regarding neck lengths, head sizes, and acetabular liner configurations, allowing the surgeon to fine tune the components chosen for final implantation with the goal of providing the patient with optimum stability and ROM.

Many in vitro and computational studies have reported ROM at impingement [14, 17]. We measured the angle at dislocation. The angle at impingement and the angle at dislocation were not the same. Bartz et al. investigated the range of flexion of the hip joint from impingement to frank dislocation, and reported that it was between 2.1° to 7.3° [18]. Robinson et al. reported 12° of joint motion from the point of impingement until dislocation was simulated [14]. Thus, the IR angles we measured were slightly larger than the IR angles measured at impingement in computational and experimental studies.

This study has several limitations. The first limitation is related to the surgical approach and posterior capsule repair. It has been reported that the surgical technique of capsular and external rotator repair had a significant effect on reducing the dislocation rate when a posterolateral approach was used [7, 22]. The dislocation rate in this study was at the upper limit of dislocation rates among recent studies [22, 23], and the dislocation rate in this study may have been related to our use of a posterolateral approach without posterior capsular or external rotator repair.

The second limitation is that this study investigated only posterior instability. Although posterior dislocation is recognized as the most common instability mode and is reported to account for 75% to 90% of dislocations [24], this study did not investigate anterior instability. Although recent study reported the factors related with anterior instability [25], more studies are needed to investigate this limitation.

The third limitation of this study is that rigid orientation standards were not employed, in that the exact position of the pelvis in space relative to the operating room floor was not available. And the repeatability of IR angle measurements and accuracy test have not been reported. To investigate the repeatability of IR angle measurements, the IR angle was measured three times in three patients during surgery. The intraobserver reproducibility was calculated using interclass correlation coefficient. The intraobserver reproducibility was excellent with 1.0 (95%CI 0.8–1.0). Because the measurements of IR angle in this study were done by the single surgeon and the same assistant surgeon, the interobserver reliability was not investigated. And many clinical studies have stated IR angle as an intraoperative stability test [4, 6, 9], and in vivo, computational, and cadaver studies have measured IR angles [8, 12]. We investigated differences in intraoperative ROM in patients without dislocation compared to patients with dislocation. As a result, we believe the IR angle to be suitable for the objectives of this study.

The fourth limitation is related to the number of dislocations and the number of the variables in this study. Peduzzi et al. reported the validity of the logistic model became problematic when the ratio of the numbers of events per variable analysed became small [26]. We performed logistic regression using three variables that were statistically significant in a chi-square test or a nonparametric Mann-Whitney U test, and using all eight variables. Logistic regression analyses determined that significant risk factors were same in both analyses; the presence of cerebral dysfunction, history of previous hip surgery, and IR angle. So, we think logistic regression analyses in this study are robust.

Despite numerous case series in the literature documenting lower dislocation rates in association with enhanced soft- tissue repair techniques and the use of large diameter femoral heads, hip instability/dislocation was one of the most common causes of revision in large series reports [1, 2]. Additional research is necessary to understand the current causes of THA instability and to enhance stability following THA.

## Conclusions

This study investigated the relationship between intraoperative stability tests and the incidence of dislocation after THA. Intraoperative stability testing, especially the IR angle, was a useful method to predict posterior stability after THA, and a larger intraoperative ROM reduced the likelihood of dislocation after THA. Cerebral dysfunction and a history of previous hip surgery are also risk factors for the incidence of dislocation after THA. Although different angles have been indicated as an acceptable ROM, 51° was indicated as a cutoff point for IR angle, and 67° was also indicated as the severe criteria of cutoff point for IR angle.

## Abbreviations

95%CI: 95% Confidence interval
AUC: Area under curve
IR angle: The range of internal rotation with 90° hip flexion
OR: Odds ratio
ROC curve: Receiver-operating characteristic curve
ROM: Range of motion
THA: Total hip arthroplasty
